# ASSOCIATION OF FLEXIBILITY WITH SOCIODEMOGRAPHIC FACTORS, PHYSICAL ACTIVITY, MUSCLE STRENGTH, AND AEROBIC FITNESS IN ADOLESCENTS FROM SOUTHERN BRAZIL

**DOI:** 10.1590/1984-0462/;2019;37;2;00005

**Published:** 2019-01-07

**Authors:** Tiago Rodrigues de Lima, Priscila Custódio Martins, Mikael Seabra Moraes, Diego Augusto Santos Silva

**Affiliations:** a Universidade Federal de Santa Catarina. Florianópolis, SC, Brasil.

**Keywords:** Healthy lifestyle, Pliability, Muscle strength, Exercise, Physical fitness, Comportamentos saudáveis, Flexibilidade, Força muscular, Atividade física, Aptidão física

## Abstract

**Objective:**

To identify sociodemographic, physical activity, and physical fitness factors associated with flexibility.

**Methods:**

Cross-sectional study with 909 adolescents (486 girls) aged 14 to 19 years from the city of São José, Santa Catarina, Brazil. To evaluate flexibility, we used the sit and reach test. Sociodemographic and physical activity data were collected by a self-administered questionnaire. We analyzed muscle strength with manual dynamometry. Aerobic fitness was assessed with the modified Canadian aerobic fitness test, and physical activity with a questionnaire. We used multiple linear regression to identify the variables associated with flexibility, with a significance level of 5%.

**Results:**

For each additional centimeter in the girls’ levels of flexibility, the boys were 2.94 cm less flexible. In addition, the increment of 0.12 kg/force in muscle strength levels increased the levels of flexibility in 1 cm.

**Conclusions:**

Lower levels of flexibility were associated with male adolescents and with reduced strength levels.

## RESMO

**Objetivo:**

Identificar os fatores sociodemográficos, de atividade física e da aptidão física associados à flexibilidade.

**Métodos:**

Estudo transversal com 909 adolescentes (486 meninas) de 14 a 19 anos da cidade de São José, Santa Catarina, Brasil. A flexibilidade foi avaliada por meio do teste de sentar e alcançar. Os dados sociodemográficos e de atividade física foram levantados por meio de questionário autoadministrado. A força muscular foi analisada por meio da dinamometria manual. A aptidão aeróbia foi analisada por meio do teste canadense modificado de aptidão aeróbia, enquanto a atividade física foi avaliada por questionário. Utilizou-se regressão linear múltipla para identificar as variáveis associadas à flexibilidade, com nível de significância de 5%.

**Resultados:**

Verificou-se que a cada centímetro a mais nos níveis de flexibilidade nas meninas, os meninos tiveram 2,94 cm a menos de flexibilidade. Ademais, o incremento de 0,12 kg/força nos níveis de força muscular aumentou em 1 cm os níveis de flexibilidade.

**Conclusões:**

Menores níveis de flexibilidade estiveram associados aos adolescentes do sexo masculino. Além disso, menores níveis de flexibilidade foram diretamente associados aos adolescentes que apresentavam menores níveis de força.

## INTRODUCTION

Flexibility is necessary to perform daily work and recreational activities.^1^ In children and adolescents, low levels of flexibility were directly associated with a higher prevalence of spinal musculoskeletal injuries, such as hyperlordosis, hyperkyphosis, and chronic low back pain.^2^ ^,^ ^3^

Given the importance of good levels of flexibility for health, different elements have been studied to explore the factors associated with this condition.^4^ ^,^ ^5^ ^,^ ^6^ Due to biological aspects, girls have higher concentrations of estrogen and collagen fibers when compared to boys, which results in greater joint and muscle range, leading to higher levels of flexibility.^7^ ^,^ ^8^ Additionally, studies have identified a direct relationship between levels of flexibility and age group among adolescents, with higher levels of flexibility in girls aged 11 to 13 years and boys aged 14 to 15 years, when compared to their peers from a lower age group, respectively.^4^ ^,^ ^6^ ^,^ ^8^ The reason for these differences is attributed to maturational development, which affects boys later than girls.^8^

In addition to biological aspects, the socioeconomic status of the family is associated with the physical fitness of children and adolescents. Low family income and low maternal schooling were related to reduced levels of flexibility. Low income can reduce the access to physical activity environments, and low maternal schooling can result in mothers knowing less about the importance of encouraging children to adopt healthy lifestyle habits, such as practicing physical activity regularly and decreasing the period of sedentary behavior.^5^ ^,^ ^9^

The regular practice of physical activity is associated with adequate levels of flexibility since physically active adolescents tend to have higher skeletal muscle, tendon, and ligament elasticity, mostly when they engage in activities that require a greater range of motion, such as sports and body expression.^1^ Variables of physical fitness are also related to lower levels of flexibility in adolescents, e.g., overweight, and low levels of aerobic fitness and muscle strength.^10^ ^,^ ^11^ ^,^ ^12^ Overweight contributes to the increase in fat tissue concentration around the joints, which decreases the range of motion and flexibility.^4^ Individuals with low levels of aerobic fitness tend to have less mechanical capacity and muscle plasticity, resulting in lower levels of flexibility.^10^ ^,^ ^13^ Also, low muscle strength levels are associated with reduced joint and muscle range.^12^

Considering the evidence of the influence of flexibility on health, identifying subgroups of adolescents more prone to this condition becomes relevant, as it could encourage preventive and health promotion actions to minimize expenses caused by the treatment of health damage associated with low levels of flexibility.^2^ Other studies^4^ ^,^ ^6^ ^,^ ^14^ investigated these constructs separately, not allowing us to assume that, in an adjusted analysis model, the associations would remain. Thus, the objective of this study was to identify the sociodemographic, physical activity, and physical fitness factors related to flexibility in adolescents from a city in Southern Brazil.

## METHOD

This school-based epidemiological research with cross-sectional design was part of the project “Brazilian guide for evaluation of physical fitness related to health and lifestyle habits - stage 1”, funded by the National Council for Scientific and Technological Development (*Conselho Nacional de Desenvolvimento Científico e Tecnológico* - CNPq). We conducted the study in 2014 in the city of São José, Santa Catarina, Brazil. The Committee for Ethics in Research with Human Beings of the Universidade Federal de Santa Catarina (CEPSH-UFSC) approved this study, under the protocol no. 746,536. Only adolescents who returned the Informed Consent Form and the Agreement Form signed participated in the research.

The population of this study comprised 5,182 high school students aged 14 to 19 years, attending state public schools in the city of São José, distributed in 11 eligible schools (stratification) and 170 classes (clustered into school hours and class/year).

Two stages established the sampling process: the first consisted of school density stratification (size: small, with less than 200 students; medium, with 200 to 499 students; and large, with 500 students or more), and the second considered school hours and class.

To determine the sample size, we chose to use the calculation for finite populations proposed by Luiz and Magnani^15^, adopting a confidence level of 1.96 (confidence interval of 95% - 95%CI), tolerable error of five percentage points, prevalence of 50% (unknown outcome), and design effect of 1.5; adding 20% to minimize potential losses and refusals to participate in the study, and other 20% to control possible confounding variables. Based on this calculation, the sample would need 751 adolescents. Due to the cluster sampling, all students were invited to participate in the study, resulting in data collected from 1,132 students. Out of them, 909 had their levels of flexibility measured and assessed (sit and reach test). Once we performed the sample calculation to determine the number of students to be interviewed by analyzing relationships that considered sample size, we calculated the statistical power for possible associations between the dependent variable and all exposure variables. The present study identified enough statistical power (value equal to or greater than 80%) to test associations between flexibility and other variables of interest.^16^

The dependent variable was flexibility, assessed by the sit and reach test. The procedure was standardized as established in the literature.^17^ We conducted the test twice and considered the highest value. The analyses used this variable continuously.

The sociodemographic variables were gender (male/female); age, in complete years, categorized into 14/15, 16/17, and 18/19 years; family income; and maternal schooling. To define the economic level, we used the questionnaire proposed by the Brazilian Association of Research Companies - *Associação Brasileira de Empresas de Pesquisa* ABEP (2010) -, which estimates the purchasing power of families. Social classes were characterized in descending order according to purchasing power (A1, A2, B1, B2, C1, C2, D, and E), based on the accumulation of material goods, housing conditions, number of domestic workers, and schooling of the head of the family.^18^ Family income was collected and subsequently categorized into up to two times the minimum wage (low); two to ten times the minimum wage (average); and more than ten times the minimum wage (high). We stratified the maternal schooling data - in complete years - into up to eight years of schooling and nine or more years of schooling, in order to match the average number of school years of Brazilian adults (7.8 years).^19^

The global physical activity variable was assessed using a version translated and validated for Brazil of the questionnaire Youth Risk Behavior Surveillance System (YRBSS)^20^ used in the United States.^21^ The question asked to evaluate global physical activity was: “During the past seven days, on how many days were you physically active for at least 60 minutes? (considering physical activity of moderate and/or vigorous intensity)”. We categorized the answers into “does not meet the recommendations” (zero to four days) and “meets the recommendations” (five or more days).^22^

A Sanny^®^ (São Paulo, Brazil) stadiometer with tripod measured the height, and a G-tech^®^ (Zhongshan, China) digital scale, the weight. Based on these variables, we calculated the body mass index (BMI). The analysis used this variable continuously.

Aerobic fitness was measured using the modified Canadian Aerobic Fitness Test - mCAFT,^17^ validated in comparison to the indirect calorimetry in Canadian men and women aged 15 to 69 years.^23^ The adolescents had to complete one or more 3-minute stages each (going up and down a double step stool with increased intensity) in predetermined rhythms according to gender and age. The test ended only when the participant reached 85% of the maximum heart rate,^17^measured with the Polar^®^ (Kempele, Finland) H7 Bluetooth heart rate sensor. The Canadian test battery determined the oxygen consumption and reference values of aerobic fitness. The equation to calculate the aerobic fitness score was: *Score=10 [17.2+(1.29 x oxygen consumption)-(0.09 x weight in kg)-(0.18 x age in years)]*. The analyses used this variable continuously.

A Saehan^®^ (Seoul, South Korea) manual dynamometer measured the handgrip strength (HGS). The test was performed twice in both hands alternately, with the result expressed in kilograms per force (kg/f); the best one of each hand was recorded and added to obtain the total force.^17^ The analyses used this variable continuously.

The adolescents self-assessed their sexual maturation, using maturational development boards. These boards had photographs of the five stages of maturational development, and the adolescents were asked to look closely at each image and mark in the questionnaire which one most resembled the size of their genitalia for boys, and breasts for girls.^24^

We applied descriptive and inferential statistics of data, verifying their normality by comparing mean and median, asymmetry*,* kurtosis, and graphs. Continuous variables were described by mean, and standard deviation. To confirm a possible difference between flexibility and the variables studied, we used Student’s *t* test and one-way ANOVA test. The effect size was calculated. For variables with two categories, the effect size was calculated with Cohen’s d test, which classifies values below 0.3 as small; lower than 0.7 as medium; and greater than or equal to 0.8 as large.^25^ For variables with three categories, we calculated the effect size using the ETA test, which considers values higher than 0.5 very large; between 0.25 and 0.5, large; between 0.05 and 0.25, medium; and lower than or equal to 0.05, small.^25^

Simple and multiple linear regression verified sociodemographic, physical activity, and physical fitness variables related to flexibility, presenting the results as regression coefficients (β) and 95%CI. In the implementation of adjusted linear regression models, we included exposure variables regardless of the p-value from the crude analysis. Interactions between independent variables were tested, but none was identified. The modeling used the backward selection method, and the criterion adopted for the permanence of the factor in adjusted models was a p-value lower than 20%. The statistical significance for association was set at 5%. We used sexual maturation as a control variable for the entire model, regardless of its p-value. The strategy used to evaluate the final model (containing variables associated with the outcome with a p-value lower than 0.05 in the adjusted analysis) was to compare several parameters (adjusted determination coefficient, regression coefficients, Akaike and Bayesian information criteria) with a saturated model (including interactions between all independent variables) and a null model (without independent variables). These assessments identified that the variables included in the final model were adjusted among themselves and in relation to the outcome. The residues of the final multiple linear regression model were evaluated by heteroscedasticity and normality. The possible multicollinearity of predictor variables of the model was investigated by the variance inflation factor (VIF). All analyses used the software Stata 12.0 (StataCorp, College Station, Texas, United States).

## RESULTS

Among the 1,132 students under study, we excluded 223 (19.7%) from the analyses as they did not perform the flexibility test, resulting in a sample of 909 students.

Out of the total of individuals evaluated, most were females (54.2%), aged 16/17 years (57.4%), had a monthly family income of two to ten times the minimum wage (68.3%), and low maternal schooling (56.2%). Approximately eight out of every ten participants did not meet the recommendations for physical activity (77.2%). The mean BMI was 22.2±3.8 kg/m^2^; the average aerobic score and muscle strength were, respectively, 388.1±58.3 and 55.3±19.0 kg/f (Table 1). Regarding gender, the mean values of the aerobic score (426.8±53.5; 353.4±36.7) and muscle strength (68.9±17.1 kg/f; 43.2±10.3 kg/f) were higher for boys when compared to girls, respectively (data not described in tables). There was a difference between average flexibility and mean values of BMI, aerobic fitness, and muscle strength of the students assessed (Table 1).

**Table 1:** Sample distribution, mean values, standard deviation, and comparison between averages of flexibility related to independent variables among students from state public schools of São José, Santa Catarina, Brazil.

	Flexibility (cm)				
	N	Sample	Mean±SD	p-value	Cohen’s d
Total	909		29.1±7.6		
Gender (%, 95%CI)					
Female*	486	54.2 (44.0-64.0)	29.5±7.5	0.081^a^	0.106
Male	423	45.8 (36.0-56.0)	28.7±7.6		
Age (years) (%, 95%CI)					
14-15	271	31.8 (30.3-33.2)	29.1±7.9	0.565^b^	0.002*
16-17	532	57.4 (55.1-59.6)	29.2±7.5		
18-19	106	10.8 (9.0-13.0)	28.8±7.5		
Income (minimum wage)^c^ (%, 95%CI)					
More than ten times the minimum wage	34	4.8 (1.7-12.4)	30.7±7.5	0.913^b^	0.018*
Two to ten times the minimum wage	516	68.3 (65.0-71.5)	29.2±7.4		
Up to two times the minimum wage	201	26.9 (19.7-35.6)	29.1±7.6		
Maternal schooling^d^ (%, 95%CI)					
9 or more years	390	43.8 (30.3-58.3)	28.9±7.6	0.378^a^	-0.052
0-8 years	507	56.2 (41.7-69.7)	29.3±7.6		
Global physical activity^e^ (%, 95%CI)					
Meets the recommendations	210	22.8 (19.4-26.6)	28.9±7.5	0.152^a^	0.013
Does not meet the recommendations	674	77.2 (73.4-80.6)	29.8±7.7		
BMI (kg/m^2^) (mean, SD)	909	22.2±3.8	-	<0.001^a^	
Aerobic fitness (aerobic score) (mean±SD)	879	388.1±58.3	-	<0.001^a^	
Muscle strength (kg/f) (mean±SD)	909	55.3±19.0	-	<0.001^a^	

n: sample; cm: centimeter; SD: standard deviation; 95%CI: confidence interval of 95%; %: percentage; BMI: body mass index; kg/m^2^: kilograms per square meter; kg/f: kilograms per strength; ^a^Student’s *t* test for independent samples; ^b^one-way ANOVA; ^c^variable with 158 missing data; ^d^variable with 12 missing data; ^e^variable with 25 missing data; *ETA^2^ test.

Table 2 presents the linear regression coefficients of crude and adjusted analyses of factors associated with flexibility. In the crude analysis, these factors were gender and muscle strength. The adjusted analysis for the variables gender and muscle strength showed that for each additional centimeter in the girls’ levels of flexibility, the boys were 2.94 m less flexible. In addition, the increment of 0.12 kg/f in muscle strength levels increased the levels of flexibility in 1 cm. The final flexibility model identified a determination coefficient of 0.0321, indicating that approximately 3.2% of the variation of flexibility was associated with the variables gender and muscle strength simultaneously. The verification of the VIF did not identify multicollinearity of variables included in the final model (VIF=1.69) (Table 2).

**Table 2:** Crude and adjusted linear regression analysis of factors associated with flexibility, and sociodemographic, physical activity, and physical fitness variables in adolescents from state public schools of São José, Santa Catarina, Brazil.

	Crude analysis			Adjusted analysis^¶^		
	ß^a^	% (95%CI)	p-value	ß^a^	% (95%CI)	p-value
Gender (male)*	-0.92	(-1.65- -0.90)	0.032	-2.94	(-4.57- -1.31)	0.016
Age (years)^†^						
16-17	0.12	(-1.59-1.85)	0.184	0.12	(-0.21-0.49)	0.289
18-19	-0.36	(-1.34-0.60)		-0.69	(-2.38-0.99)	
Income (minimum wage)^‡^						
Two to ten times	-1.46	(-9.07-6.16)	0.704	-0.83	(-7.21-5.55)	0.942
Up to two times	-1.48	(-10.77-7.80)		-0.53	(-8.70-7.63)	
Maternal schooling (0-8 years)^§^	-0.43	(-3.77-2.90)	0.631	-0.52	(-5.31-4.25)	0.681
Global physical activity (Does not meet the recommendations)^||^	-0.81	(-3.14-1.51)	0.270	-0.25	(-2.97-2.46)	0.723
BMI (kg/m^2^)	0.10	(-0.35-0.57)	0.894	-0.17	(-0.02-0.47)	0.894
Aerobic fitness (aerobic score)	-0.04	(-0.03-0.21)	0.891	-0.02	(-0.01-0.01)	0.891
Muscle strength (kg/f)	0.03	(0.01-0.06)	0.028	0.12	(0.06-0.13)	0.007

%: percentage; 95%CI: confidence interval of 95%; ^a^regression coefficient; *reference value compared to females; ^†^reference values compared to the category 14-15 years; ^‡^reference values compared to the category more than ten times the minimum wage; ^§^reference values compared to the category 9-11 years; ^||^reference values compared to the category meets the recommendations; BMI: body mass index; k/m^2^: kilograms per square meter; kg/f: kilograms per force. All variables were included in the adjusted model regardless of the p-value from the crude analysis. Variables with p≥0.20 were excluded from the adjusted model. The final model, with gender and muscle strength variables, showed: R2=0.0321; AIC=4829.59; BIC=4811.38; VIF=1.69; and F=333.61

## DISCUSSION

The main findings of this study indicated that children and adolescents with lower muscle strength levels had reduced levels of flexibility. Also, for each additional centimeter in the girls’ levels of flexibility, the boys were 2.94 cm less flexible. In addition, the increment of 0.12 kg/f in muscle strength levels increased the levels of flexibility in 1 cm.

Thus, the present study revealed that, in comparison to girls, boys had reduced levels of flexibility. The literature presents similar results.^1^ ^,^ ^26^ A study carried out with students from a city in Southern Brazil did not identify a significant difference in flexibility regarding gender.^4^ The differences found among the results of several studies could be due to the sample used since other variables can act as confounding factors, such as genetics,^27^ age^2^, and level of physical activity.^5^ However, a large part of the scientific literature describes differences between genders in levels of flexibility of adolescents.^2^

Boys tend to have reduced levels of flexibility from adolescence to adulthood.^8^ The reason for this is that the progression of maturation stages can compromise them in tests that evaluate flexibility, considering that, concomitantly with their maturational development, there is an increase in the production of anabolic hormones directly associated with muscle hypertrophy and lower density of muscle and ligament tissues, which can lead to less flexible muscles.^28^ Moreover, differences in body structures, such as lower tissue density, in addition to more elastic and flexible ligaments and muscles give girls a greater range of motion.^7^ Also, cultural aspects can contribute to the disparity in levels of flexibility concerning gender, as during adolescence, boys tend to engage in activities that require a more evident use of skeletal muscles and subsequent development of muscle strength.^1^ Girls, on the other hand, tend to participate in activities that emphasize movements of flexibility, such as dances.^26^

In the present study, adolescents with lower muscle strength levels showed reduced values of flexibility. A study conducted in the Netherlands, whose sample consisted of 548 pairs of twin adolescents aged 16 to 19 years, did not identify an association between muscle strength (measured by dynamometry) and flexibility (sit and reach test).^27^ A possible justification for the interrelationship between lower muscle strength levels and reduced levels of flexibility might be connected to neuromuscular aspects caused by muscle fiber hypertrophy.^12^ The development of skeletal muscles - responsible for increased muscle strength - can improve the interaction between actin and myosin filaments, and increase capillarity and oxidative capacity, factors that can contribute to a better flexibility.^28^ In addition, lower strength levels are associated with the increase in tendon stiffness, negatively related to the degree of flexibility and joint motion.^29^

The present study found no association of flexibility with family income and maternal schooling. The lack of this association can be explained by the transitions that happened in the 21st century, mainly regarding changes in the lifestyle of the population.^9^ A significant part of mothers is away from home the whole day, in long work hours, and, therefore, the time they spend with their children is shorter, which can lead to less time to guide them on healthy life habits.^11^ Thus, a large number of adolescents tend to accumulate extended hours of sedentary behavior and little time practicing physical activities, particularly those that require a greater range of motion.^2^ ^,^ ^3^

This research did not identify an association between flexibility and physical activity. The lack of statistical significance could be justified, in part, by the fact that approximately 50% of the variability in levels of flexibility is attributed to the genetic component^27^; therefore, biological characteristics would have a greater influence on levels of flexibility than behavioral ones.^26^ Moreover, the use of instruments that directly measure physical activity, such as accelerometers, could reduce the response bias from the individuals assessed. The present study did not use this form of evaluation, which probably contributed to the lack of association between physical activity and flexibility.^13^

 The present study found no association of flexibility with BMI and aerobic fitness. A possible explanation for the lack of association between flexibility and BMI could be the inability to evaluate the components of body composition separately when using this index, considering that it does not allow the identification of fat, muscle, or bone mass distribution, and the influence of these components on flexibility.^10^ The lack of association between flexibility and aerobic fitness could be justified by the type of physical activity that results in an improvement of this physical fitness component. Aerobic activities and those of greater intensity promote better physical performance and increase the levels of VO_2_max, favoring the transport of oxygen from the lungs to the tissues; however, these aspects are not directly associated with levels of flexibility and, therefore, might not provide direct benefits to them.^30^

 The limitations of the study include data collection with a self-administered questionnaire since it could result in response bias regarding the identification of variables such as sociodemographic factors and level of physical activity. Nonetheless, the questionnaire used was validated for the population evaluated. Another limitation was relying on self-report to identify sexual maturation. Although magnetic resonance imaging is the reference method to determine the maturational development in adolescents,^16^ aspects related to the environment suitable for installation and the high cost of equipment precludes its use in studies with a large number of participants. Furthermore, the cross-sectional nature of the study does not allow us to establish a causal relationship between variables.

This study contributes to the health sector, as it identified subgroups of adolescents who should be prioritized in strategies to increase levels of flexibility. These strategies can be developed in the school environment with activities to improve muscle strength, using exercises that stimulate the overload of the body and higher joint range and motion, such as dances. Moreover, we underline the use of sexual maturity as a control variable in the analysis as a strength of the study, given that the changes caused by the pubertal period can influence the physical fitness tests in adolescents.^13^

We concluded that male adolescents and those with lower strength levels had reduced levels of flexibility.
